# Role of Neuromuscular Electrical Stimulation in Increasing Femoral Venous Blood Flow After Total Hip Prosthesis

**DOI:** 10.7759/cureus.29255

**Published:** 2022-09-17

**Authors:** Murat Calbiyik, Seyhan Yılmaz

**Affiliations:** 1 Orthopaedics and Traumatology, Hitit University Faculty of Medicine, Corum, TUR; 2 Cardiovascular Surgery, Amasya University Faculty of Medicine, Amasya, TUR

**Keywords:** nmes device, femoral vein, hip prosthesis, venous thrombosis, electrical stimulation

## Abstract

Objective: This study aimed to investigate the role of neuromuscular electrical stimulation (NMES) in increasing femoral venous blood flow after total hip prosthesis and to evaluate its potential effects on preventing postoperative deep vein thrombosis (DVT).

Materials and methods: A total of 64 patients who underwent total hip prosthesis were randomly separated into two groups. The NMES group (n=32) received low-molecular-weight heparin+NMES. And the non-NMES group (n=32) received a low-molecular-weight heparin+compression bandage.

Results: There was no difference between the groups in terms of the presence of preoperative and postoperative leg edema. The calf diameter was significantly lower in the NMES group than in the non-NMES group in both the preoperative (p=0.003) and postoperative (p=0.008) period. Although the femoral vein peak velocity (VPV) was similar between the groups in the preoperative period, it was significantly higher in the NMES group than in the non-NMES group postoperatively (p=0.001). The femoral VPV after total hip prosthesis increased more in the NMES group (43.2%) compared with the non-NMES group (16.3%). In the non-NMES group, the D-dimer value in the preoperative period was lower than on postoperative days one and five (p<0.05). There was no significant difference between the D-dimer values on postoperative days one and five. In the NMES group, a statistically significant difference was determined between the preoperative and postoperative test results (F(2.93)=20.86, p=0.001). The preoperative D-dimer values were compared to the postoperative values on the first and fifth day, and according to the post hoc test results, the D-dimer values were significantly lower on the fifth postoperative day than on the first postoperative day, and the preoperative value was significantly lower than the fifth postoperative day value (p<0.05).

Conclusion: Although the two groups were similar in terms of leg edema, there was a significant increase in femoral VPV in the NMES group. This could indicate a potential effect of NMES in preventing postoperative DVT and needs to be confirmed with further studies.

## Introduction

In the early postoperative period, patients undergoing orthopedic surgery are at risk of deep vein thrombosis (DVT) and pulmonary embolism, which increase morbidity and mortality, prolonged hospital stay, and health expenditure [1,2]. Venous stasis due to long-term immobility is the main cause of DVT. For patients undergoing orthopedic surgery, regulating hemodynamic processes and increasing venous blood flow during the postoperative recovery period in the hospital are essential in preventing DVT. Pharmacological and non-pharmacological interventions are performed for this purpose [3]. A wide range of non-pharmacological modalities, including cryotherapy, continuous passive motion, surface electromyographic biofeedback, and shockwave therapy, can be used to regulate hemodynamic processes [3].

Neuromuscular electrical stimulation (NMES) has been used as an auxiliary method to improve body functions in various rehabilitation programs [4]. In this method, superficial skeletal muscles are regularly stimulated via electrodes placed on the skin over the relevant muscle, and visible tetanic contractions are created. Thus, muscular atrophy is also prevented in patients who are immobile for a long time due to trauma, surgery, or illness [5]. As a beneficial method of preventing and managing venous diseases, NMES improves venous hemodynamic parameters, such as peak velocity and volume flow [6]. The hemodynamic and muscle-strengthening effects of NMES (i.e., function-improving effects of NMES during postoperative and rehabilitation periods) have been investigated in various patient groups with orthopedic problems and/or who have undergone orthopedic surgery [7-9].

The aim of this study was to investigate the role of NMES in increasing femoral venous blood flow after total hip prosthesis and to evaluate its potential effect on preventing postoperative DVT.

## Materials and methods

Study design and population

This prospective study was initiated following the approval (approval no. 14KAEK-108) of the local Ethics Committee and was conducted in accordance with the latest version of the Declaration of Helsinki. The sample size was calculated by power analysis based on the work of Broderick et al. [10]. It was calculated that at least 12 patients in each group would provide 95% of the sample power. Participants were briefed on the content of the study and signed informed consent forms with a detailed description of the study procedures.

According to the criteria-based sampling method, a total of 64 patients were eligible. Inclusion criteria for patients were a diagnosis of primary coxarthrosis and having undergone total hip replacement surgery at the Gaziosmanpasa University Training and Research Hospital between September 2016 and October 2020. The study exclusion criteria were defined as lower extremity surgery due to previous trauma, multiple trauma related to femur or tibia fracture, history of varicose veins, severe obesity, congestive heart failure, venous thromboembolism (VTE), family history of VTE, previous knee arthroplasty, diabetes mellitus, smoking, kidney failure, severe motor neuron disease, ankle-brachial index <0.9, malignancy and/or coagulopathy, myocardial infarction, cardiac arrhythmia, percutaneous coronary or cardiac intervention undergoing stent or pacemaker implantation, systolic blood pressure >180 mmHg and <100 mmHg, diastolic blood pressure >100 mmHg, and cardiac rhythm disturbances.

Working procedures and groups

All patients were operated on in the lateral Sim's position while under regional anesthesia by the same surgeon using a modified Gibson incision. After surgery, the patients were randomly divided into two groups using a list of random numbers and the sealed envelope method. Immediately before surgery, an envelope was opened by a staff member not involved in the study, and the patient was allocated to either the NMES or non-NMES group. Low molecular weight heparin plus NMES was applied to the NMES group (n=32), while low molecular weight heparin plus compression bandage was applied to the non-NMES group (n=32).

Low molecular weight heparin was administered to the patients at a dose of 4,000 anti-XA IU/0.4 mL for two weeks. A 20 cm compression bandage was wrapped around the leg up to the hip joint at moderate pressure. Cefazolin was administered prophylactically 90 minutes before surgery at a dose of 1 g for patients <80 kg, 2 g for patients >80 kg, and 3 g for patients >120 kg, with repeat dose intervals of six to eight hours for a maximum of 24 hours. A hemovac drain placed during surgery was removed on the first postoperative day.

**Figure 1:**
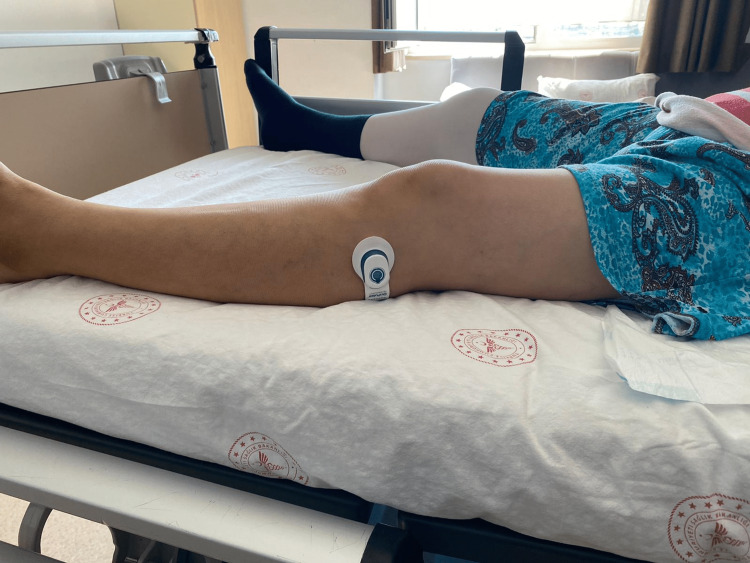
Patient wearing the Geko™device

Neuromuscular electrical stimulation was performed in accordance with the standardized protocol. The NMES device (Geko™, First Kind Ltd., High Wycombe, UK) was placed by the orthopedist at the end of the fourth hour postoperatively to stimulate the common peroneal nerve, and electrostimulation was provided at 1 Hz, pulse 70-560, and 27 mA according to the manufacturer's instructions. The Geko T-1 device was applied according to the manufacturer’s instructions. The device was worn on the leg 24 hours a day during the acute phase (day one to six) to prevent VTE (Figure 1). The NMES device was changed daily in accordance with the manufacturer’s instructions for use. A nurse was assigned to check if the device was functioning well for one hour every four hours during the ward stay. The patients were allowed to mobilize out of bed on the day after the operation and encouraged to walk during their hospital stay. The device was operated only while the patients were resting in bed lying in the supine position. The NMES device was removed on discharge.

The demographic data of the patients were recorded. The presence of leg edema before and after the operation was investigated with the calf diameter measured 7 cm above the medial malleolus by the physiotherapist. The D-dimer levels were measured in the preoperative period and on the first and fifth days postoperatively. Preoperatively and on the fifth postoperative day, the femoral vein blood flow rate was measured by Doppler ultrasonography (USG) of the lower extremities to investigate the development of DVT. Ultrasonography was performed on each patient in a supine position during inspiration with the foot in 10 degrees plantar flexion by a radiologist blinded to the patient groups.

Statistical analysis

Data were analyzed using Statistical Package for Social Sciences (SPSS) version 22.0 (IBM Corp., Armonk, NY, USA). Conformity of data to normal distribution was analyzed using Kolmogorov-Smirnov and Shapiro-Wilk tests. Descriptive statistics were presented as mean ± standard deviation values ​​for continuous variables, numbers, and percentages for categorical data. Categorical variables were compared using the chi-square test. The Student's t-test was used to compare normally distributed numerical measurements between two independent groups, and the Mann-Whitney U-test was used for non-normally distributed measurements. Paired t-test was used to compare normally distributed numerical measures between two dependent groups, and the Wilcoxon signed-rank test was used for non-normally distributed measures. A comparison of numerical measures between three independent groups was analyzed with the one-way analysis of variance (ANOVA) test. A p-value of <0.05 was accepted as the statistical significance level.

## Results

The general characteristics of the patients in the study with and without postoperative NMES are listed in Table 1. Both groups were similar in terms of age, gender, and body mass index.

**Table 1:** General characteristics of the study patients The values are presented as mean±standard deviation values or number and percentage. a: Mann-Whitney U-test, b: Chi-square test, c: Student’s t-test, NMES: Neuromuscular electrical stimulation

		NMES group (n=32)	Non-NMES group (n=32)	p-value
Age (years) (x̄±sd)		62.0±14.2	61.5±12.4	0.874^a^
Gender n (%)	Male	15 (46.9)	16 (50)	0.802^b^
	Female	17 (53.1)	16 (50)	
Body mass index (x̄±sd)		27.95±1.38	28.42±1.62	0.216^c^

The preoperative and postoperative characteristics of the patients are presented in Table 2. None of the patients had clinical symptoms or ultrasound findings of postoperative vein peak velocity. Overall, the length of hospital stay and the frequency of leg edema before and after surgery were similar in the two groups. Calf diameter showed a significant increase after surgery compared to the preoperative period in both groups. Calf diameter was significantly lower in the NMES group (mean (m)=38.38, standard deviation (s)=3.38) than in the non-NMES group (m=40.88, s=3.51 cm, p=0.003) preoperatively, and postoperatively (m=39.00 s=3.17; m=41.84 s=4.39, p=0.008, respectively) as well. The femoral vein peak velocity (VPV) in the preoperative period was similar in both groups and was determined to be significantly higher in the NMES group than in the non-NMES group postoperatively (m=15.59, s=2.08; m=10.78 s=3.48 cm/s, p=0.001). After total hip replacement, femoral VPV increased by 16.3% in the non-NMES group and by 43.2% in the NMES group.

**Table 2:** Preoperative and postoperative characteristics of the patients The values are presented as mean±standard deviation values or number and percentage. a: Chi-square test, b: Mann-Whitney U test, c: Wilcoxon signed-rank test, NMES: Neuromuscular electrical stimulation

			NMES group (n=32)	Non-NMES group (n=32)	P-value	
Leg edema (%)	Preoperative	17 (53.1)		14 (43.8)		0.453^a^
	Postoperative		21 (65.6)	18 (56.3)	0.442^a^	
	P		0.309^a^	0.317^a^		
Calf diameter (cm) (x̄±sd)	Preoperative		38.38±3.38	40.88±3.51	0.003^b^	
	Postoperative		39.00±3.17	41.84±4.39	0.008^b^	
	P		0.001^c^	0.003^c^		
Femoral vein peak velocity (cm/s) (x̄±sd)	Preoperative		8.86±1.55	9.02±2.68	0.791^b^	
	Postoperative		15.59±2.08	10.78±3.48	0.001^b^	
	P		<0.001^c^	0.066^c^		
Postoperative hospital stay (days) (x̄±sd)			7.0±1.34	7.5±1.8	0.355^b^	

The independent samples t-test was performed to compare the D-dimer levels of the patients in the non-NMES and NMES groups (Table 3). Preoperatively, no significant difference was determined at the t=1.70 p=0.094 level in the D-dimer values between the NMES group (m=0.71, s=0.67) and the non-NMES group (m=1.01, s=0.69). On postoperative day one, there was no significant difference in D-dimer values at the t=0.276 p=0.783 level between the non-NMES group (m=2.86, s=1.46) and the NMES group (m=2.75, s=1.73). On postoperative day five at t=2.603 p=0.012 level, the D-dimer level of the NMES group (m=1.68, s=1.15) was significantly lower than in the non-NMES group (m=2.43, s=1.17). These results show that the application of NMES is effective in reducing the D-dimer level on the fifth postoperative day.

**Table 3:** Comparisons of the mean D-Dimer values of the groups preoperatively, and on postoperative day one and day five. Independent samples t-test p<0.05* x̄:mean, sd: standard deviation

	Non-NMES		NMES			
D-Dimer	x̄	sd	x̄	sd	t	p
Preoperative	1.01	0.69	0.71	0.67	1.70	0.094
Postoperative day 1	2.86	1.46	2.75	1.73	0.276	0.783
Postoperative day 5	2.43	1.17	1.68	1.15	2.603	0.012*

The paired samples t-test was used to determine whether there was a statistically significant difference between the levels of two repeated measurements. The mean value of the NMES group on postoperative day one1 was higher than the mean value preoperatively and on postoperative day 5 five, and the mean value on the fifth day after surgery was higher than the preoperative value (p<0.005). The same results were obtained in the non-NMES group (Table 4).

**Table 4:** Comparisons of the mean D-Dimer preoperatively, and on postoperative day one and five. Paired T-test p<0.05* x̄:mean, sd: standard deviation

	D-Dimer		x̄	sd	t	p
	Preoperative (1)	1-2	1.01	0.69	1-2:-6.80	0.001*
Non-NMES	Postoperative day 1 (2)	2-3	2.86	1.46	2-3:-3.17	0.003*
	Postoperative day 5 (3)	1-3	2.43	1.17	1-3:-6.30	0.001*
NMES	Preoperative (1)	1-2	0.71	0.67	1-2:-7.39	0.001*
	Postoperative day 1 (2)	2-3	2.75	1.73	2-3:4.44	0.001*
	Postoperative day 5 (3)	1-3	1.68	1.15	1-3:-5.50	0.001*

According to the ANOVA results, a statistically significant difference was found between the test averages of the non-NMES group (F(2.93)=22.63, p=0.001). The Tukey honestly significant difference (HSD) post hoc pairwise comparison test was used to determine from which group the difference originated. According to the post-hoc test results, the preoperative D-dimer mean value was significantly lower than the mean value on postoperative days one and five (p<0.005). There was no significant difference between the mean D-dimer values on postoperative days one and five. A statistically significant difference was found between the test averages of the NMES group (F(2.93)=20.86, p=0.001). According to the post hoc test results, the mean preoperative D-dimer value was significantly lower than the mean values of the postoperative day one and five, and the preoperative mean value was significantly lower than the postoperative day five mean value (p<0.005) (Table 5).

**Table 5:** Comparisons of the mean D-Dimer values preoperatively, and on postoperative day one and five. Analysis of variance (ANOVA) test p<0.05* x̄:mean, sd: standard deviation

		x̄	sd	p	Post-hoc p-value
	Preoperative (1)	1.01	0.69	0.001*	1-2:0.001*
Non-NMES	Postoperative day 1 (2)	2.86	1.46		2-3:0.298
	Postoperative day 5 (3)	2.43	1.17		1-3:0.001*
NMES	Preoperative (1)	0.71	0.67	0.001*	1-2:0.001*
	Postoperative day 1 (2)	2.75	1.73		2-3:0.003*
	Postoperative day 5 (3)	1.68	1.15		1-3:0.009*

## Discussion

Growing evidence shows that NMES performed as an auxiliary treatment method after orthopedic surgery enhances muscle strength and has positive effects on improving function [11-16]. It has been demonstrated that NMES can also be safely performed in patients with a metal hip/knee implant [17,18]. The NMES procedure also enhances venous, arterial, and microvascular blood flow [19], and it has been suggested that NMES may be beneficial in reducing edema due to its microcirculation-improving effect [20]. Nevertheless, in the present study, none of the results confirms the edema-reducing effect of NMES but a significant increase in the femoral VPV was recorded only in the NMES group.

In this study, the effect of electrical stimulation of the common peroneal nerve on femoral venous blood flow was evaluated using an NMES device (Geko ™) after total hip prosthesis surgery. The Geko ™ device is a battery-powered and disposable tool that is intended to reduce the risk of VTE [21]. It has been suggested that performing NMES in addition to pharmacological prophylaxis may be beneficial in reducing the incidence of DVT in patients undergoing orthopedic surgery [18,22]. In a systematic review evaluating the role of NMES in thromboprophylaxis, Hajibandeh et al. concluded that NMES increased venous blood flow and was well tolerated, although current evidence does not promote the role of NMES in thromboprophylaxis. Furthermore, it was emphasized that randomized clinical trials are required on this subject [23]. Increased blood flow does not indicate increased flow, although contraction of the calf muscle may increase velocity, but not necessarily increase overall flow. The use of the peak venous rate for the incidence of VTE is controversial because there is no evidence that a high peak venous rate results in a lower incidence of DVT [24]. It has also been argued that a high peak velocity-inducing device can increase DVT [25]. Again, it has been emphasized that there is a need for randomized clinical studies on this subject.

During total knee arthroplasty (TKA), excessive flexion and dislocation of the knee and swelling of the thigh under tourniquet may cause direct trauma to the vasculature of the operated leg and platelet adhesion according to Virchow's triad. Sharrock et al. [26] showed increases in systemic circulation thrombosis indices (D-dimer) immediately after tourniquet removal, and transesophageal echocardiography studies have also detected embolism during tourniquet inflation and following deflation [27,28]. Although the exact incidence of VPV during TKA is unknown, Maynard et al. [29] reported that 87% of patients with asymptomatic DVT were already positive within one day after surgery. All these reports indicated that there is a definite risk of VPV during TKA. Prevention of venous stasis and blood hypercoagulation by thromboprophylactic transcutaneous nerve stimulation (TENS) has the potential to prevent such an abnormal state during TKA.

In a pilot study of patients who underwent total knee replacement, Yilmaz et al. found that postoperative femoral vein peak flow velocity was significantly higher among patients who received NMES (n=15, m=17.46, s=2.86)compared with those who did not receive it (n=15, m=13.84, s=3.58 cm/s, p=0.02) [22]. The mean femoral vein peak flow velocity increased by 67.48% with the postoperative NMES application compared with the preoperative values. Broderick et al. evaluated five patients who underwent total hip arthroplasty and reported that performing NMES in the early postoperative period showed favorable hemodynamic effects [18]. It was suggested that NMES would be beneficial in preventing DVT and reducing edema. Nevertheless, the requirement for the confirmation of the results in studies with larger sample sizes was emphasized. Broderick et al. demonstrated that NMES applied to the calf muscle of patients undergoing total hip arthroplasty (n=10) or total knee arthroplasty (n=10) increased the peak venous velocity by 200% and the mean velocity of the popliteal vein by 60%, which were measured by Doppler ultrasound, compared with the resting values [18]. Another study by Broderick et al. demonstrated that NMES applied to the calf muscle of patients undergoing total hip replacement (n=11) increased the peak venous velocity by up to 99% and the mean velocity by 178%, which were measured by popliteal venous Doppler ultrasound, compared with the resting values [10].

In addition to the common factors that contribute to the formation of DVT such as venous stasis, vascular endothelial injury, and blood hypercoagulability, the hypothesis of “the fourth thrombotic factor” has also been suggested in recent years [30]. This hypothesis suggests that there are innervating nerves around the vein, which can induce periodic changes in the diameter of blood vessels or have a direct antithrombotic effect through neurohumoral action. This mode of action of local electrical stimulation of NMES may also enhance the efficacy in preventing the formation of DVT.

The peak time for DVT to appear is one to 14 days after an operation and peak formation occurs in about three days, so early detection and prevention are of paramount importance [31]. Nikolaev et al. performed USG on postoperative day seven [32], Xiong et al. on day five [33], and Izumi et al. on postoperative day one [34].

In recent years, NMES has been widely used in the prevention of DVT. The mechanism of effect is that the electric current stimulates the regular impulses of lower limb nerves, causing the rhythmic contraction of lower limb muscles, increasing the pump function of muscles, and effectively improving the circulatory state of the venous and lymphatic system of lower extremities [21]. At the same time, after the improvement of local circulation, the metabolism of the wound site will also be accelerated, which can effectively reduce the local aggregation of procoagulant substances, reduce the reactive adhesion of platelets, and reduce the hypercoagulable state of blood [31]. This process also promotes the excretion of local inflammatory mediators and reduces local inflammatory reactions, swelling, and postoperative pain. It also accelerates the metabolic rate of plasma D-dimer without increasing the negative pressure drainage after surgery [19].

The coagulation function of the patient effectively reflects the hypercoagulable or fibrinolytic state of blood in the human body with plasma D-dimer content. When the hyperfibrinolysis and thrombosis procedure in the body and the plasma D-dimer content increases, the formation of DVT can be easily induced. In this study, there was a significant difference in plasma D-dimer content between the two groups on the fifth postoperative day. The NMES administration can effectively reduce plasma D-dimer content after an operation. Considering the above-mentioned results, it was observed that the NMES reduced DVT formation.

In the current study of patients who underwent total hip prosthesis, postoperative bandage application was found to increase the femoral VPV by 78.8%, and the NMES procedure by 66.8%. Similarly, the femoral VPV was significantly higher in the NMES group (m=15.59, s=2.08) than in the non-NMES group (m=10.78, s=3.48cm/s, p<0.001) during the postoperative period. While the femoral VPV increased by only 16.3% in the non-NMES group, the NMES procedure induced a 43.2% increase in the femoral VPV after total hip prosthesis.

The fact that NMES increases deep venous blood flow in the lower extremities has also been demonstrated in some studies involving healthy subjects. In a study by Griffin et al. common peroneal nerve stimulation was performed with the Geko ™ device, with significant increases reported in velocity and flow volume in response to electrical stimulation in all three veins [35]. Peak velocity increased by 216% in the peroneal vein, 112% in the posterior tibial vein, and 137% in the gastrocnemius vein. In another study of healthy subjects, Warwick et al. provided stimulation using the Geko ™ device and reported improved microcirculation in the foot. The response was seen to be greater when lying down and non-weight bearing than weight bearing standing, but the most striking effect was when stimulation was combined with a plaster cast [36].

Limitation

Although the outcomes of this study can be generalized to both genders, the age of the participants was a limiting factor in the study. All the patients in the study were aged > 60 years since the younger population does not need hip surgery under normal conditions other than accidents or extreme trauma. Therefore, frequency, pulse width, amplitude, and the use of variable frequency pulse patterns may vary in younger patients who have higher muscle tonus and power. Modification of any of these parameters might also affect the recovery of older patients who may vary in muscle strength and/or subcutaneous tissue thickness and should therefore be adjusted for each patient. Surface-stimulating electrodes direct the current precisely beneath the surface area of the electrode and this current travels through various viscosities of subcutaneous tissue which create resistance that limit the depth of penetration depending on the subcutaneous tissue thickness. Standardization of pulse frequency, pulse width, amplitude, and electrode size should be standardized for subcutaneous tissue thickness in future studies. Another limitation was the lack of an additional control group receiving standard rehabilitation alone.

## Conclusions

The results of this study showed a beneficial hemodynamic response to NMES for the prevention of DVT and the reduction of edema in patients in the early postoperative period following orthopedic surgery.

Not all VTEs occur in the early postoperative period, although there is no doubt that surgery is the 'trigger' of perioperative VTE. In this context, thromboprophylactic NMES would seem to be a rational tool to prevent subsequent adverse events. It was concluded that the use of NMES in postoperative orthopedic patients can be considered a DVT prevention method.

Evaluation of the potential of NMES to prevent DVT in combination with other thromboprophylaxis modalities is an area for further research. It would also be beneficial to investigate the optimal parameters of NMES (intensity, frequency, duration, etc.), and stimulation location to provide more meaningful references.

## References

[1] Saleh J, El-Othmani MM, Saleh KJ (2017). Deep vein thrombosis and pulmonary embolism considerations in orthopedic surgery. Orthop Clin North Am.

[2] Rahman A, Colak MC, Ustünel L (2009). A comparison of different treatment managements in patients with acute deep vein thrombosis by the effects on enhancing venous outflow in the lower limb. Med Sci Monit.

[3] Gatewood CT, Tran AA, Dragoo JL (2017). The efficacy of post-operative devices following knee arthroscopic surgery: a systematic review. Knee Surg Sports Traumatol Arthrosc.

[4] Veldman MP, Gondin J, Place N, Maffiuletti NA (2016). Effects of neuromuscular electrical stimulation training on endurance performance. Front Physiol.

[5] Spector P, Laufer Y, Elboim Gabyzon M, Kittelson A, Stevens Lapsley J, Maffiuletti NA (2016). Neuromuscular electrical stimulation therapy to restore quadriceps muscle function in patients after orthopaedic surgery: a novel structured approach. J Bone Joint Surg Am.

[6] Williams KJ, Ravikumar R, Gaweesh AS (2017). A review of the evidence to support neuromuscular electrical stimulation in the prevention and management of venous disease. Adv Exp Med Biol.

[7] Boucher T, Wang S, Trudelle-Jackson E, Olson S (2009). Effectiveness of surface electromyographic biofeedback-triggered neuromuscular electrical stimulation on knee rehabilitation. N Am J Sports Phys Ther.

[8] Lepley LK, Wojtys EM, Palmieri-Smith RM (2015). Combination of eccentric exercise and neuromuscular electrical stimulation to improve quadriceps function post-ACL reconstruction. Knee.

[9] Bremner CB, Holcomb WR, Brown CD, Perreault ME (2017). The effectiveness of neuromuscular electrical stimulation in improving voluntary activation of the quadriceps: a critically appraised topic. J Sport Rehabil.

[10] Broderick BJ, Breathnach O, Condon F (2013). Haemodynamic performance of neuromuscular electrical stimulation (NMES) during recovery from total hip arthroplasty. J Orthop Surg Res.

[11] Kim KM, Croy T, Hertel J, Saliba S (2010). Effects of neuromuscular electrical stimulation after anterior cruciate ligament reconstruction on quadriceps strength, function, and patient-oriented outcomes: a systematic review. J Orthop Sports Phys Ther.

[12] Walls RJ, McHugh G, O'Gorman DJ, Moyna NM, O'Byrne JM (2010). Effects of preoperative neuromuscular electrical stimulation on quadriceps strength and functional recovery in total knee arthroplasty. A pilot study. BMC Musculoskelet Disord.

[13] Hasegawa S, Kobayashi M, Arai R, Tamaki A, Nakamura T, Moritani T (2011). Effect of early implementation of electrical muscle stimulation to prevent muscle atrophy and weakness in patients after anterior cruciate ligament reconstruction. J Electromyogr Kinesiol.

[14] Stevens-Lapsley JE, Balter JE, Wolfe P, Eckhoff DG, Kohrt WM (2012). Early neuromuscular electrical stimulation to improve quadriceps muscle strength after total knee arthroplasty: a randomized controlled trial. Phys Ther.

[15] Kittelson AJ, Stackhouse SK, Stevens-Lapsley JE (2013). Neuromuscular electrical stimulation after total joint arthroplasty: a critical review of recent controlled studies. Eur J Phys Rehabil Med.

[16] Levine M, McElroy K, Stakich V, Cicco J (2013). Comparing conventional physical therapy rehabilitation with neuromuscular electrical stimulation after TKA. Orthopedics.

[17] Broderick BJ, Kennedy C, Breen PP, Kearns SR, Olaighin G (2010). The influence of orthopaedic implants on patient tolerance of neuromuscular electrical stimulation (NMES). Annu Int Conf IEEE Eng Med Biol Soc.

[18] Broderick BJ, Kennedy C, Breen PP, Kearns SR, ÓLaighin G (2011). Patient tolerance of neuromuscular electrical stimulation (NMES) in the presence of orthopaedic implants. Med Eng Phys.

[19] Tucker A, Maass A, Bain D, Chen LH, Azzam M, Dawson H, Johnston A (2010). Augmentation of venous, arterial and microvascular blood supply in the leg by isometric neuromuscular stimulation via the peroneal nerve. Int J Angiol.

[20] Bahadori S, Immins T, Wainwright TW (2017). The effect of calf neuromuscular electrical stimulation and intermittent pneumatic compression on thigh microcirculation. Microvasc Res.

[21] Summers JA, Clinch J, Radhakrishnan M (2015). The Geko™ electro-stimulation device for venous thromboembolism prophylaxis: a NICE medical technology guidance. Appl Health Econ Health Policy.

[22] Yilmaz S, Calbiyik M, Yilmaz BK, Aksoy E (2016). Potential role of electrostimulation in augmentation of venous blood flow after total knee replacement: a pilot study. Phlebology.

[23] Hajibandeh S, Hajibandeh S, Antoniou GA, Scurr JR, Torella F (2015). Neuromuscular electrical stimulation for thromboprophylaxis: a systematic review. Phlebology.

[24] Morris RJ, Woodcock JP (2004). Evidence-based compression: prevention of stasis and deep vein thrombosis. Ann Surg.

[25] Proctor MC, Greenfield LJ, Wakefield TW, Zajkowski PJ (2001). A clinical comparison of pneumatic compression devices: the basis for selection. J Vasc Surg.

[26] Sharrock NE, Go G, Sculco TP, Ranawat CS, Maynard MJ, Harpel PC (1995). Changes in circulatory indices of thrombosis and fibrinolysis during total knee arthroplasty performed under tourniquet. J Arthroplasty.

[27] Kato N, Nakanishi K, Yoshino S, Ogawa R (2002). Abnormal echogenic findings detected by transesophageal echocardiography and cardiorespiratory impairment during total knee arthroplasty with tourniquet. Anesthesiology.

[28] Berman AT, Parmet JL, Harding SP (1998). Emboli observed with use of transesophageal echocardiography immediately after tourniquet release during total knee arthroplasty with cement. J Bone Joint Surg Am.

[29] Maynard MJ, Sculco TP, Ghelman B (1991). Progression and regression of deep vein thrombosis after total knee arthroplasty. Clin Orthop Relat Res.

[30] Nikolaev KN, Ivchenko DR, Akimov AV (2020). Electric muscle stimulation for prevention of venous thromboembolism in patients with multiple lower extremity trauma. Traumatology and Orthopedics of Russia.

[31] Tian W (2016). Guidelines for prevention of venous thromboembolism in major orthopedic surgery in China. Chin J Orthop.

[32] Xiong J, Zhang Q, Li Y (2022). Clinical study of neuromuscular electrical stimulation in the prevention of deep venous thrombosis of lower extremities after anterior cruciate ligament reconstruction. J Healthc Eng.

[33] Izumi M, Ikeuchi M, Aso K (2015). Less deep vein thrombosis due to transcutaneous fibular nerve stimulation in total knee arthroplasty: a randomized controlled trial. Knee Surg Sports Traumatol Arthrosc.

[34] Stefanou C (2016). Electrical muscle stimulation in thomboprophylaxis: review and a derived hypothesis about thrombogenesis—the 4th factor. Springerplus.

[35] Griffin M, Bond D, Nicolaides A (2016). Measurement of blood flow in the deep veins of the lower limb using the Geko™ neuromuscular electro-stimulation device. Int Angiol.

[36] Warwick D, Shaikh A, Worsley P (2015). Microcirculation in the foot is augmented by neuromuscular stimulation via the common peroneal nerve in different lower limb postures: a potential treatment for leg ulcers. Int Angiol.

